# Enhanced Piezoelectric Properties in a Single-Phase Region of Sm-Modified Lead-Free (Ba,Ca)(Zr,Ti)O_3_ Ceramics

**DOI:** 10.3390/ma15217839

**Published:** 2022-11-07

**Authors:** Andong Xiao, Xuefan Xie, Liqiang He, Yang Yang, Yuanchao Ji

**Affiliations:** Frontier Institute of Science and Technology and State Key Laboratory for Mechanical Behavior of Materials, Xi’an Jiaotong University, Xi’an 710049, China

**Keywords:** lead-free ceramics, single ferroelectric phase, enhanced piezoelectric effect, *c/a* ratio, miniaturized domains

## Abstract

In ferroelectric materials, phase boundaries such as the morphotropic phase boundary (MPB) and polymorphic phase boundary (PPB) have been widely utilized to enhance the piezoelectric properties. However, for a single-ferroelectric-phase system, there are few effective paradigms to achieve the enhancement of piezoelectric properties. Herein, we report an unexpected finding that largely enhanced piezoelectric properties occur in a single-tetragonal-ferroelectric-phase region in the Sm-modified (Ba_0.85_Ca_0.15_)(Zr_0.1_Ti_0.9_)O_3_ (BCZT-*x*Sm) system. An electrostrain maximum (0.13%) appears in the single-phase region of the BZCT-0.5Sm composition with the maximum polarization (*P*_m_ = 18.37 µC/cm^2^) and piezoelectric coefficient (*d*_33_ = 396 pC/N) and the minimum coercive field (*E*_C_ = 3.30 kV/cm) at room temperature. Such an enhanced piezoelectric effect is due to the synergistic effect of large lattice distortion and domain miniaturization on the basis of the transmission electron microscope (TEM) observation and X-ray diffraction (XRD) Rietveld refinement. Our work may provide new insights into the design of high-performance ferroelectrics in the single-phase region.

## 1. Introduction

Piezoelectric materials have been widely used in numerous electromechanical applications such as sensors, transducers, and nano-positioners, representing a huge market valued at approximately USD 25 billion [1,2,3]. Since the discovery of Pb(Ti,Zr)O_3_ (PZT) in the 1950s, the piezoelectric material market has been dominated by lead-based ceramics, such as Pb(Ti,Zr)O_3_-based (PZT-based) or Pb(Mg,Nb)O_3_-PbTiO_3_-based (PMN-PT-based) ceramics, due to their high piezoelectric and ferroelectric properties [4]. However, with increasing environmental concerns about the toxicity of lead, it has become critical to develop lead-free piezoelectric materials with similar excellent performance over a wide temperature range to replace commercial lead-based ceramics in numerous electronic devices [5].

For more than sixty years, ferroelectric phase boundaries such as the morphotropic phase boundary (MPB) and polymorphic phase boundary (PPB) have been widely utilized in lead-based and lead-free piezoelectric systems to achieve ferroelectric ceramics with great piezoelectric and ferroelectric properties [6,7,8,9,10,11,12,13,14]. At the phase boundary, the instability of the polarization state caused by the phase transition between the several kinds of ferroelectric phases leads to easy rotation of the polarization directions under external electric or stress fields [15,16,17], and the miniaturized hierarchical domain structure of the composition located in ferroelectric phase boundaries induces low-energy barriers to domain wall motion [18,19,20,21]. As a result, such a composition can achieve a high piezoelectric coefficient and electrostrain [22,23,24,25]. As the composition moves away from the phase boundary, the coexisting ferroelectric phases transform into a single ferroelectric phase, resulting in the stabilized polarization states and micro-sized domain structures [18]. The enhanced energy barriers of polarization rotation and domain wall motion largely reduce the piezoelectric properties in a single-ferroelectric-phase region [1]. As a result, there is a rarely effective pathway to enhance the piezoelectric properties by modifying a single ferroelectric phase.

Here, surprisingly, we found enhanced piezoelectric properties in the single tetragonal ferroelectric phase in Sm-doped (Ba_0.85_Ca_0.15_)(Zr_0.1_Ti_0.9_)O_3_ (BCZT-*x*Sm, where *x* is the molar percent of Sm dopants) ceramics, differing from the modified piezoelectric properties at the phase boundary by the introduction of Sm in previous studies [26,27,28]. The optimal composition of the BCZT-0.5Sm ceramics exhibits an enhanced piezoelectric coefficient *d*_33_ and electrostrain and maximum polarization over a wide temperature range compared with the adjacent samples with the same tetragonal phase. XRD refinement by the Rietveld method and transmission electron microscope results show that the BCZT-0.5Sm ceramics have a large *c*/*a* ratio and miniaturized domains, which may be responsible for the enhanced properties of this material.

## 2. Experimental Procedure

((Ba_0.85_Ca_0.15_)_1–1.5x/100_Sm_x/100_)(Zr_0.1_Ti_0.9_)O_3_ (BCZT-*x*Sm, *x* = 0, 0.25, 0.5, 0.75, 1, and 1.5, where *x* is the molar percentage of Sm) ceramics were prepared by a conventional solid-state reaction route with BaCO_3_ (99.8%, Alfa Aesar), TiO_2_ (99.8%, Alfa Aesar), BaZrO_3_ (99%, Alfa Aesar), CaCO_3_ (99.5%, Alfa Aesar), and Sm_2_O_3_ (99.99%, Alfa Aesar) powders. After complete drying, the precursors were weighed and then ball-milled in alcohol with a zirconium oxide milling ball and Nylon jar for 6–8 h. The powders were calcined at 1350 °C for 3 h after drying. The mixture was ball-milled for a second time for 6–8 h, then dried, granulated with 5 wt.% polyvinyl alcohol (PVA) solution, and compacted into pellets at 120 MPa. The green pellets were sintered at 1450 °C for 5 h. Sliver paste was painted on both sides of the pellets, and the pellets were heated at 700 °C for 30 min.

The crystal structure of the samples was characterized by X-ray diffraction (XRD) using a Shimadzu XRD7000 from 20° to 85° at a step of 0.02° and a rate of 2°/min with CuKα radiation at a constant current of 30 mA and voltage of 40 kV. The refined spectrum of XRD was measured at a step of 0.01° and a rate of 0.6°/min. A field emission scanning electron microscope (Quanta 250 FEG) was used to reveal the surface morphology of the BCZT-*x*Sm ceramics at an acceleration voltage of 10 kV. The density of all samples was measured by the Archimedes method with the medium of water. The relative density of all samples was above 96%. The temperature dependence of the dielectric permittivity (*ε*_r_) was measured from 150 °C to −100 °C by a HIOKI LCR meter at a cooling rate of 2 °C/min (Delta temperature chamber). The dielectric spectroscopy oscillation voltage was 1 V, and the LCR frequency was 1 kHz, 10 kHz, and 100 kHz. Polarization-electric field (*P*-*E*) loops and electrostrain loops were tested at 10 Hz and 30 kV/cm by a Premier Ⅱ ferroelectric test system and MTI2100 measurements. The cylinder specimens with silver paste were poled at 25 °C under a DC field of 1 kV/mm for half an hour, and the temperature dependences of the piezoelectric coefficient d33 values were measured by a piezoelectric d33-meter (Model ZJ-3B, Chinese Academy of Sciences) with a self-made temperature chamber (in a silicon oil bath), which relied on collector magnetic stirrer (LANSGT 101S) warming and liquid nitrogen cooling. The local microstructural evolution and corresponding diffraction patterns were obtained by a JEOL-2100F high-resolution transmission electron microscope (TEM) equipped with a double-tilt heated sample stage at an acceleration voltage of 200 kV and analyzed by the Digital Micrograph software.

## 3. Results and Discussion

### 3.1. Sample Characterization and Microstructure

**Figure 1:**
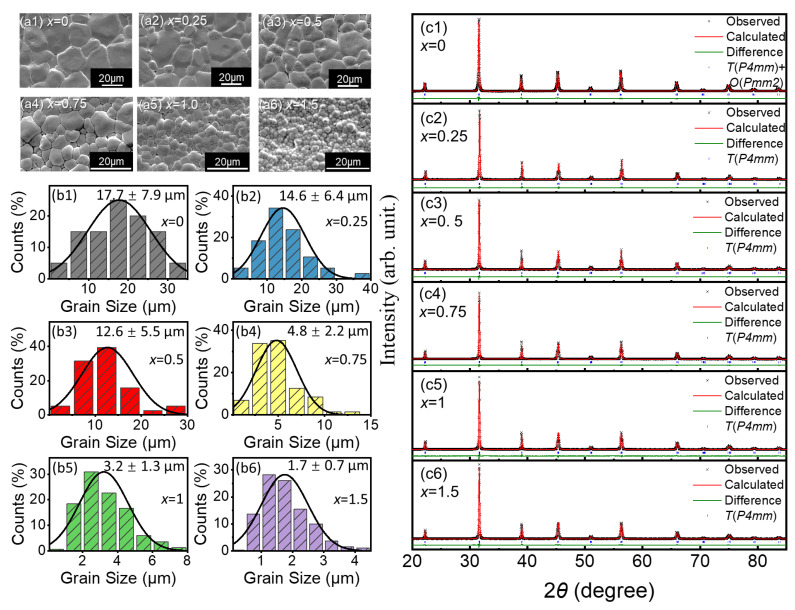
(**a1**–**a6**) SEM images and (**b1**–**b6**) normal distribution diagrams of the grain size statistics of BCZT-*x*Sm (*x* = 0, 0.25, 0.5, 0.75, 1 and 1.5) ceramics; mean values and standard deviations are marked in the statistics diagram. XRD patterns and the Rietveld refinement of the XRD data for BCZT-*x*Sm with (**c1**) *x* = 0, (**c2**) *x* = 0.25, (**c3**) *x* = 0.5, (**c4**) *x* = 0.75, (**c5**) *x* = 1, and (**c6**) *x* = 1.5.

Figure 1a shows the surface morphology of the BCZT-*x*Sm ceramics. All samples had a densely packed microstructure with a uniform grain size distribution. The normal distribution of the grains was obtained by the statistics of the grain size in the SEM images by the ImageJ software, and the means and standard deviations of the grain sizes were also obtained, as shown in Figure 1b. With the introduction of Sm, the grain size gradually decreased from 17.7 μm (*x* = 0) to 1.7 μm (*x* = 1.5). The decreasing trend of the grain sizes is reported to be related to the decrease of the grain boundary mobility after doping [29].

From the XRD patterns of different Sm-doped ceramics, it can be seen that the crystal structure was a pure perovskite structure without any secondary phase in the range of 20° to 85° [26,27,28,30]. Figure 1c shows the XRD Rietveld refinement results of the BCZT-*x*Sm (*x* = 0, 0.25, 0.5, 0.75, 1, and 1.5) ceramics by the GSAS-EXPGUI program, which were well fit to the observed data. The refinement of the diffraction patterns showed that all Sm modified samples were in the ferroelectric tetragonal (*T*) phase with the *P4mm* space group at room temperature [31]. In addition, the reliability factors (*Rp*), error factors (*χ^2^*), and lattice parameters are listed in Table 1. The low *Rp* (%) and *χ^2^* (%) of the samples illustrate the reliability of the refined XRD results. As shown in Table 1, when the doping amount of Sm was 0.5, the lattice distortion (*c/a* ratio) was the highest (1.0046), while it was 1.0041 for BCZT-0.25Sm and 1.0042 for BCZT-0.75Sm.

Rietveld refinement of these modified BCZT-Sm XRD results showed that the *c/a* ratio increased and then decreased sharply, which may be affected by the electronegativity and ionic radius of Sm. In addition, by calculating the tolerance factor, as shown in the following equation, it can be seen from Table 2 that, with the increase of Sm ion doping content, the tolerance factor decreased continuously.

$$t=\frac{R_{A}+R_{O}}{\sqrt{2}(R_{B}+R_{O})}$$

where *R*_A_ is the effective ionic radius of the A-site, *R_B_* is the effective ionic radius of the B-site, and *R_O_* is the effective ionic radius of the O-site. As the tolerance factor became smaller, the tetragonal structure became more unstable and transformed into a cubic structure [33,34,35]. The characteristic peak splitting gradually disappeared, leading to the decrease of the *c/a* ratio.

### 3.2. Dielectric and Ferroelectric Properties at Room Temperature

**Figure 2:**
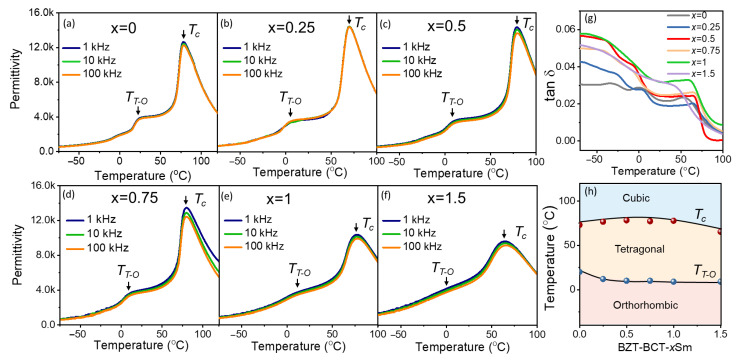
(**a**–**f**) Temperature-dependent permittivity of the BCZT-*x*Sm ceramics at different frequencies (1 kHz, 10 kHz, and 100 kHz), where *x* = 0, 0.25, 0.5, 0.75, 1, and 1.5, respectively; (**g**) the dielectric loss at 100 kHz and (**h**) corresponding temperature composition phase diagram showing the Curie temperature (*T_C_*) and the phase transition temperature (*T_T-O_*) from the tetragonal phase to the orthorhombic phase.

Figure 2a–g show the temperature-dependent permittivity curves upon cooling of BCZT-*x*Sm (*x* = 0, 0.25, 0.5, 0.75, 1, and 1.5) ceramics at different frequencies (1 kHz, 10 kHz, and 100 kHz) and the dielectric loss at 100 kHz. With the increase of the Sm concentration, the maximum permittivity at the Curie temperature gradually decreased. When the doping concentration of Sm was 1.5, the dielectric permittivity curves exhibited diffuse phase transitions from cubic to tetragonal and from tetragonal to orthorhombic, indicating a reduction in the difference between cubic symmetry and ferroelectric non-center symmetry, as well as the disruption of long-range-order ferroelectric domains. As shown in Figure 2h, with the increment of Sm, the Curie temperature (*T*_C_) gradually increased and then decreased. In addition, the temperature of the tetragonal (*T*) to orthorhombic (*O*) phase transition remained almost constant (10 °C) away from room temperature. Therefore, the BCZT-*x*Sm ceramics exhibited a ferroelectric tetragonal phase at room temperature, which was consistent with the results of the Rietveld-refined XRD.

**Figure 3:**
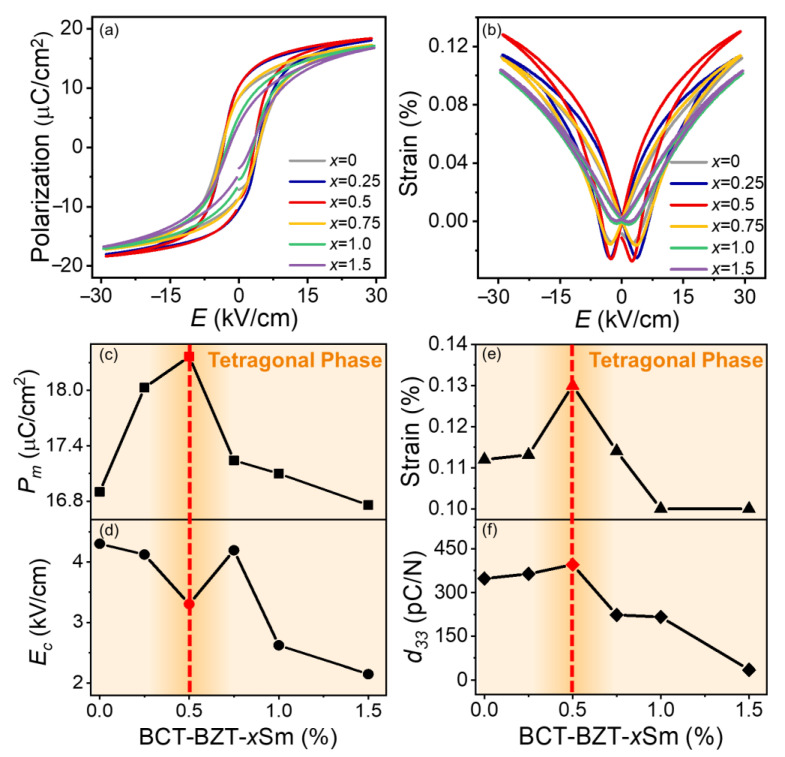
(**a**) Polarization-electric field loops and (**b**) electrostrain loops of the BCZT-*x*Sm (*x* = 0, 0.25, 0.5, 0.75, 1, and 1.5) ceramics; composition-dependent (**c**) maximum polarization (*P*_m_), (**d**) coercive field (*E*_C_), (**e**) strain, and (**f**) piezoelectric coefficient (*d*_33_) curves measured in the tetragonal-phase region.

Figure 3a,b show the polarization-electric field (*P*-*E*) loops and electrostrain loops of the unmodified BCZT and BCZT-*x*Sm ceramics at 30 kV/cm in the tetragonal-phase region, whose temperature was 10 °C higher than that of the *T*-*O* phase boundary. With increasing Sm doping concentration, the *P*-*E* loops and electrostrain curves became slim, indicating the miniaturization of the ferroelectric domains. To elucidate the changes in the ferroelectric, piezoelectric, and electromechanical properties after introducing Sm to the BCZT system, Figure 3c–f display the composition-dependent maximum polarization (*P*_m_), coercive field (*E*_C_), strain, and piezoelectric coefficient (*d*_33_) in the single tetragonal phase. As shown in Figure 3c, the BCZT-0.5Sm ceramic exhibited an enhanced effect of the piezoelectric properties, which showed a larger *P*_m_ (18.37 µC/cm^2^), strain (0.13%), and *d*_33_ (396 pC/N) and a lower *E*_C_ (3.30 kV/cm) compared with those of other ceramics. With the introduction of more Sm dopants (*x* = 1 and 1.5), the normal ferroelectric tetragonal phase gradually transformed to a relaxor state, leading to the degradation of the piezoelectric and ferroelectric properties. The above results indicated that the BCZT-0.5Sm ceramic exhibited an enhanced piezoelectric effect in a single ferroelectric phase.

### 3.3. Enhanced Piezoelectric Properties over a Wide Temperature Range

**Figure 4:**
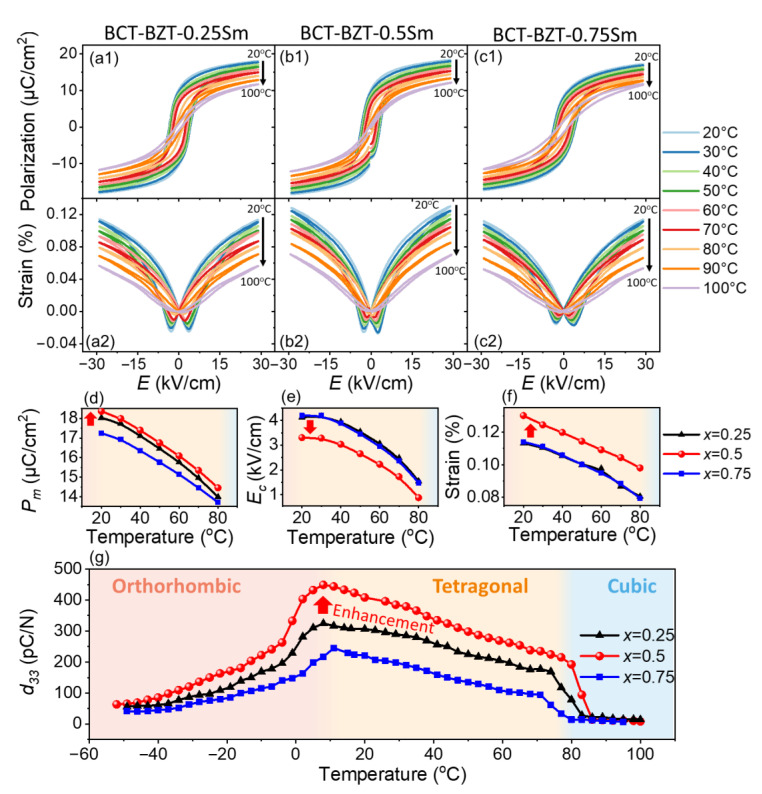
(**a1**–**c2**) The temperature-dependent polarization (*P*)-electric field (*E*) loop and strain of the BCZT-*x*Sm (*x* = 0.25, 0.5, 0.75) ceramics; temperature-dependent (**d**) maximum polarization (*P*_m_), (**e**) coercive field (*E*_C_), (**f**) strain, and (**g**) piezoelectric coefficient (*d*_33_) curves of the BCZT-*x*Sm (*x* = 0.25, 0.5, and 0.75) ceramics.

Furthermore, this enhancement effect could exist not only at room temperature, but also over a wide temperature range across the tetragonal phase. The temperature-dependent polarization-electric field (*P*-*E*) loops and electrostrain loops of the BCZT-*x*Sm (*x* = 0.25, 0.5, 0.75) ceramics from 20 °C to 100 °C are shown in Figure 4a–c. Compared with the BCZT-0.25Sm and BCZT-0.75Sm ceramics, the BCZT-0.5Sm ceramic showed larger *P*_m_ and strain and lower *E*_C_ upon heating (Figure 4d–f). Figure 4g presents the temperature-dependent piezoelectric coefficient *d*_33_ when heated from −20 °C to 100 °C. The *d*_33_ change of the BCZT-*x*Sm ceramics peaked around 10 °C, indicating the *T*-*O* phase boundary [36]. With further heating, the *d*_33_ of the BCZT-0.5Sm ceramic was much larger than that of the BCZT-0.75Sm ceramic in the single-tetragonal-phase region. It is clear from the temperature-dependent results that the enhancement effect of the BCZT-0.5Sm ceramic in a single ferroelectric phase worked over a wide range of temperatures below *T*_C_.

### 3.4. Properties’ Microstructure Relationship in a Single-Phase Region

**Figure 5:**
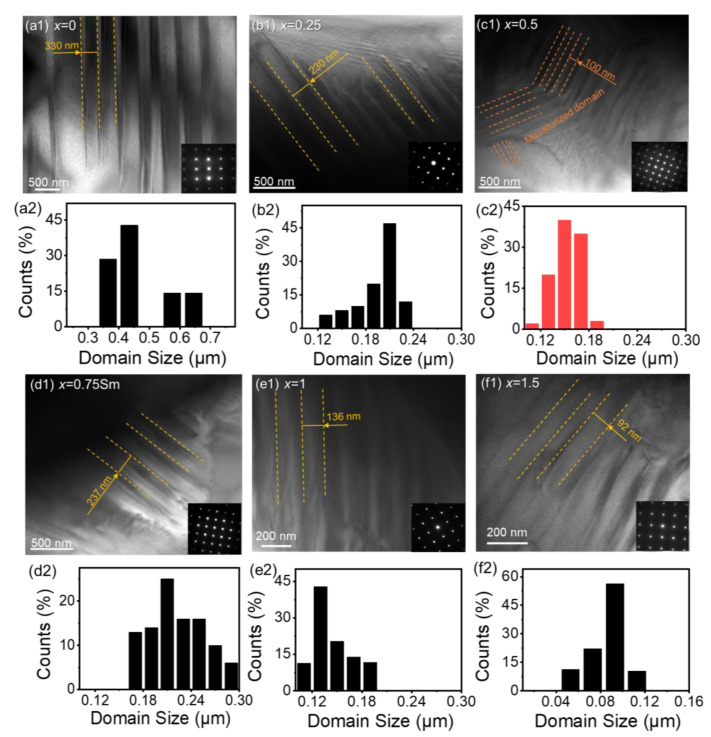
(**a1**–**f1**) Transmission electron microscope (TEM) observation of the (100) zone axis and (**a2**–**f2**) the corresponding domain size distribution of the BCZT-*x*Sm ceramics with *x* = 0, 0.25, 0.5, 0.75, 1, and 1.5, where BCZT-0.5Sm displayed unexpected miniaturized domains.

In order to reveal the enhanced piezoelectric effect of the Sm-modified BCZT ceramics, the microstructure of different compositions was probed from the (100) zone axis by transmission electron microscopy (TEM). Figure 5(a1–f1) show the ferroelectric domain structures and the corresponding average domain sizes of the BCZT-*x*Sm (*x* = 0, 0.25, 0.5, 0.75, 1, and 1.5) ceramics [31,37] in Figure 5(a2–f2), respectively. Figure 5(b1,d1) display the lamellar domain patterns of the BCZT-0.25Sm and BCZT-0.75Sm ceramics, while for the BCZT-0.5Sm ceramic, the domain pattern showed a hierarchical structure, as shown in Figure 5(c1). Compared with the average domain size of the BCZT-0.25Sm (120 nm–240 nm) and BCZT-0.75Sm (160 nm–300 nm) ceramics, the average domain size of the BCZT-0.5Sm ceramic was the lowest (100 nm–200 nm).

For ferroelectrics, the electric-field-induced strain comes from two aspects, that is the intrinsic contribution from electrostriction and the inverse piezoelectric effect and the extrinsic contribution from the domain wall motion [38,39,40,41,42]. For electrostriction, when an external electric field is applied, ions shifting away from the equilibrium sites lead to the variation of the lattice parameters and the relative strain [43,44]. Therefore, the large lattice distortion, indicating a large variation of the lattice parameter, favors the maximum polarization (*P*_m_) and piezoelectric coefficient (*d*_33_) and the intrinsic contribution of electrostriction for electric-field-induced strain [45]. On the other hand, the electrostrain is also generated by the motion of ferroelectric non-180° domain walls, accompanied by a hysteresis from the energy barrier of domain rotation [44,45]. Therefore, even though the domain sizes of BCZT-1Sm and BCZT-1.5Sm were also reduced, the XRD peak splitting was gradually less pronounced at this time, and the spontaneous polarization was reduced, resulting in a lower polarization response under an external electric field, a gradual decrease in polarization and strain, and a reduction in the notification hysteresis.

**Figure 6:**
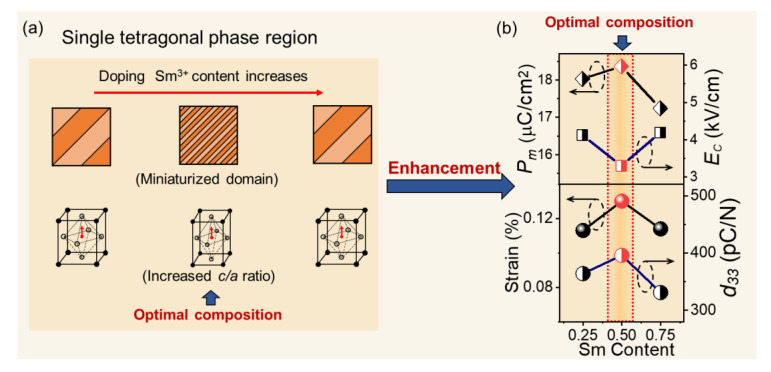
Schematic diagram of the enhanced piezoelectric effect in a single-tetragonal-phase region due to (**a**) the increased *c/a* ratio and miniaturized domain, compared with those of the adjacent compositions, showing (**b**) an increased *P*_m_, strain, and *d*_33_ and a reduced *E*_C_.

As shown in Figure 6, after introducing Sm into the BCZT ceramics, the average domain size of BCZT-0.5Sm was the smallest, indicating the flattened energy distribution for domain wall motion and a low coercive field (*E_c_*) for polarization rotation [46,47,48]. The easier domain wall movement induced by the miniaturized domain favored the maximum polarization (*P*_m_) and piezoelectric coefficient (*d*_33_) and the extrinsic contribution of electric-field-induced strain. Therefore, an enhanced piezoelectric effect was exhibited in a single-ferroelectric-tetragonal-phase region owing to the extrinsic contribution of the miniaturized hierarchical domain structure and the intrinsic contribution of the large lattice distortion.

## 4. Conclusions

In summary, the enhanced piezoelectric effect was reported in the single ferroelectric tetragonal phase of Sm-modified BCZT ceramics. The phase diagram showed that the Curie temperature firstly increased and then decreased, while the temperature of the tetragonal and orthorhombic phase transition almost kept the same (10 °C) with the increment of the Sm concentration. Compared to BZCT-0.25Sm and BZCT-0.75Sm, BZCT-0.5Sm had the largest electrostrain (0.13%), maximum polarization (18.37 µC/cm^2^), and piezoelectric coefficient (396 pC/N) and the lowest coercive field (3.30 kV/cm) at room temperature. Based on the refined XRD results and TEM observation, the BCZT-0.5Sm ceramic showed the largest lattice distortion (*c/a* ratio = 1.004737) and a miniaturized hierarchical domain structure, where the average domain size was 100 nm–200 nm. In addition, such an enhanced piezoelectric effect can be maintained over a wide temperature range from −50 °C to 75 °C, including tetragonal and orthorhombic phase ranges. Our work may provide an efficient route to design high-performance ferroelectric materials in a single-ferroelectric-phase region

## Figures and Tables

**Table 1:** Structure parameters of BCZT-*x*mol%Sm obtained by Rietveld refinement.

Composition	*Rp* (%)	Error Factor *χ^2^* (%)	Space Group	*a* (Å)	*b* (Å)	*c* (Å)	*c/a*	*α/β/γ*	Unit Cell Volume (Å^3^)
*x* = 0 [32]	7.57		*P4mm* *Pmm2*	3.971(5) 4.012(1)	3.971(5) 4.012(1)	4.007(0) 4.012(1)	1.009 1	90/90/90 89.6/89.6/89.6	63.2017 64.5845
*x* = 0 *x* = 0.25	8.78 8.91	1.88 1.98	*P4mm + Pmm2* *P4mm*	4.005(3) 3.993(6)	4.005(3) 3.993(6)	4.014(9) 4.009(7)	1.0024 1.0041	90/90/90 90/90/90	64.3526 63.9489
*x* = 0.5	7.98	1.57	*P4mm*	3.996(8)	3.996(8)	4.015(3)	1.0046	90/90/90	64.1425
*x* = 0.75 *x* = 1 *x* = 1.5	7.15 8.13 7.82	1.36 1.63 1.44	*P4mm* *P4mm* *P4mm*	3.996(8) 3.996(9) 3.997(1)	3.996(8) 3.996(9) 3.997(1)	4.013(6) 4.010(9) 4.008(0)	1.0042 1.0035 1.0027	90/90/90 90/90/90 90/90/90	64.0796 64.0749 64.0350

**Table 2:** Tolerance factors and differences for several different compositions.

Composition	*t*
*x* = 0.25	1.0408
*x* = 0.5	1.0406
*x* = 0.75 *x* = 1 *x* = 1.5	1.0393 1.0376 1.0357

## Data Availability

The data that support the findings of this study are available within this article.
